# Potential Impacts of Future Warming and Land Use Changes on Intra-Urban Heat Exposure in Houston, Texas

**DOI:** 10.1371/journal.pone.0148890

**Published:** 2016-02-10

**Authors:** Kathryn Conlon, Andrew Monaghan, Mary Hayden, Olga Wilhelmi

**Affiliations:** National Center for Atmospheric Research, Boulder, Colorado, United States of America; University of Washington, UNITED STATES

## Abstract

Extreme heat events in the United States are projected to become more frequent and intense as a result of climate change. We investigated the individual and combined effects of land use and warming on the spatial and temporal distribution of daily minimum temperature (T_min_) and daily maximum heat index (HI_max_) during summer in Houston, Texas. Present-day (2010) and near-future (2040) parcel-level land use scenarios were embedded within 1-km resolution land surface model (LSM) simulations. For each land use scenario, LSM simulations were conducted for climatic scenarios representative of both the present-day and near-future periods. LSM simulations assuming present-day climate but 2040 land use patterns led to spatially heterogeneous temperature changes characterized by warmer conditions over most areas, with summer average increases of up to 1.5°C (T_min_) and 7.3°C (HI_max_) in some newly developed suburban areas compared to simulations using 2010 land use patterns. LSM simulations assuming present-day land use but a 1°C temperature increase above the urban canopy (consistent with warming projections for 2040) yielded more spatially homogeneous metropolitan-wide average increases of about 1°C (T_min_) and 2.5°C (HI_max_), respectively. LSM simulations assuming both land use and warming for 2040 led to summer average increases of up to 2.5°C (T_min_) and 8.3°C (HI_max_), with the largest increases in areas projected to be converted to residential, industrial and mixed-use types. Our results suggest that urbanization and climate change may significantly increase the average number of summer days that exceed current threshold temperatures for initiating a heat advisory for metropolitan Houston, potentially increasing population exposure to extreme heat.

## Introduction

Exposure to high temperatures among populations living in urban areas poses a serious threat to human health [1–3]. Climate models suggest that future United States (U.S.) urban populations will disproportionately experience extreme heat [4] and possibly increased mortality [5–7], in part due to the urban heat island (UHI) effect, whereby the built environment facilitates higher air and surface temperatures in urban areas relative to surrounding suburban and exurban areas [8]. Despite growing efforts to reduce heat-related health impacts via implementing warning systems [9], increasing surveillance [10], promoting air conditioning use, establishing cooling centers, and enhancing education on heat-related health outcomes [11], heat is a substantial driver of weather-related morbidity and mortality in the U.S [12] and most often affects urban populations.

The combination of extreme heat and the UHI creates dangerous conditions for urban populations [13]. For example, between 35,000 and 70,000 and over 700 excess deaths have been attributed to heat during the 2003 European and 1995 Chicago heat waves, respectively [14–16]. Such events have brought attention to the dramatic and complex impacts that high temperatures have on human health. Elevated risk of hospitalization related to renal failure, electrolyte imbalance and respiratory illness is associated with increasing temperatures [17–19]. Degraded air quality is commonly observed in urban areas and during heat waves can further exacerbate adverse health outcomes like respiratory illness, asthma and chronic obstructive pulmonary disease [20–22]. Further epidemiological evidence indicates that certain urban subpopulations are most vulnerable to extreme heat [23–25], with much of that burden placed on elderly [26], minorities [27], those with lower socioeconomic status [28], individuals with chronic health conditions (e.g., congestive heart failure, diabetes) [29], and populations living in less vegetated neighborhoods [30].

Although quantifying exposure to extreme heat can be challenging [23], common measures include local weather station observations of daily minimum temperature and maximum heat index. The heat index is a measurement of perceived discomfort as the body cannot cool in high humidity. Heat index is calculated as a function of ambient air temperature and relative humidity [31] and is used by U.S. National Weather Service (NWS). Metrics that combine the effects of temperature and humidity have been associated with increased heat-related mortality [32], which is particularly relevant for humid cities like Houston. Minimum temperature measurements capture the heat-retaining effects of urban areas compared to their surrounding rural areas during nighttime, such that high minimum temperatures may indicate periods when overnight ambient temperatures do not drop low enough to provide respite from high daytime temperatures, which has been associated with deleterious health outcomes including mortality [25]. In addition to the urban built environment, it is noteworthy that high humidity in cities like Houston can also lead to higher nighttime minimum temperatures because water vapor reduces the loss of longwave radiation from the surface to space [33].

Local drivers of urbanization can substantially change the energy balance and reflectivity (i.e., albedo) of land, ultimately impacting the UHI [34]. Impervious surfaces, such as concrete, asphalt, and brick, have low albedos indicating that they easily absorb and retain energy from solar radiation. Vegetation loss is accompanied by diminished evapotranspiration, which in turn leads to elevated ambient temperatures because more energy is available for sensible heating [35]. Urban expansion and development often increase the impervious area and lower the vegetated fraction of the surface, leading to an increase in the UHI. Urban canopy models (UCMs) are designed to simulate these and related processes that occur within the urban canopy, the layer of air closest to the surface, extending upward to approximately the mean building height [36]. UCMs are included within land surface models (LSMs); LSMs in turn simulate the surface energy balance for a wide range of land use types, including urban [37]. LSMs need accurate land use and land cover characterizations in order to properly simulate the surface energy balance and estimate meteorological variables, like near-surface temperatures [38]. LSMs can either be run on their own [39], or coupled to atmospheric models [38, 40].

Recent studies have used coupled LSM/UCM-atmospheric model frameworks to investigate how urban landscapes impact the distribution of heat. Adachi et al. [41] simulated how the UHI of Tokyo may change due to altering the city footprint to be either more dispersed or more compact. They found that the UHI was more widespread for the dispersed development scenario, but extreme heat events were more intense in the urban core for the compact scenario. Similarly, Martilli [42] found that although denser cities consumed less energy than dispersed cities, the combination of high density and low vegetation lead to higher heat stress. Georgescu et al. [43] performed 20-km simulations over the southern and southwestern U.S. for several scenarios of projected urban growth (and accompanying land use change) for 2050 and found that warming due to urbanization may be on the same order as projected warming due to climate change, though the implementation of “cool” (high albedo) roofs could reduce the impact by about 50%. Thus, from a city planning perspective there are tradeoffs between dispersed and compact cities, and between the costs and benefits of implementing expensive mitigation measures, in terms of their effect on extent and intensity of the UHI.

Similarly, coupled LSM/UCM-atmospheric model frameworks have been used to investigate how climate change may alter urban extreme heat. Kusaka et al. [44] performed 3-km simulations for the three largest urban areas in Japan for historical and 2070s conditions assuming a moderate emissions scenario, and projected that August temperatures will be about 2.3°C warmer in the 2070s, with more than twice as many days triggering heat alerts. Argüeso, et al. [45] conducted 2-km simulations for Sydney, Australia for a 20-year period centered on 2050 for an aggressive emissions scenario and accounting for urban growth, and found that daily minimum temperatures increased substantially as a result of both urbanization and climate change. While these studies support the expectation that urbanization and climate change will increase urban temperatures, they do not incorporate planned, fine-scale land use projections into their models, which may be useful for understanding how land use change could affect the distribution of extreme heat across a metropolitan area.

With the expectation that both climate change and land use change will, over time, contribute to the intensification of UHIs in the U.S. [46], there is a need to characterize how population exposure to heat will change over time and space. The present study employs a LSM/UCM on its own (i.e., uncoupled to an atmospheric model) to simulate the impact of land use change and warming due to climate change on the UHI of Houston, Texas. Here we define land use change as being due to both urbanization (the expansion of urban areas into rural areas) and the conversion of land use from one land use type to another within pre-existing urban areas. Our simplified approach is up to several orders of magnitude less computationally expensive than using a coupled LSM/UCM-atmospheric model framework, and therefore may be a useful template for others who wish to perform similar studies but lack the computational resources. The LSM/UCM methodology follows Monaghan et al. [47], which yielded accurate results. As part of a larger, NASA-funded project entitled “System for Integrated Modeling of Metropolitan Extreme heat Risk (SIMMER)” [48], this study builds on our present, empirically-derived understanding of Houston’s UHI by incorporating parcel-level land use data to develop a better-informed model of the future distribution of extreme heat.

## Methods

### Houston, Texas

Houston, Texas is a large southern U.S. city that experiences high ambient temperatures and humidity during summer. High heat stress days and nights are projected to become more frequent in the future [49]. With a land area of 600 square miles, Houston has a metropolitan population of over 6 million as of 2014 that is estimated to reach nearly 10 million by mid-century [50]. Houston is considered one of the most sprawling cities in the U.S. (U.S. Census Bureau 2014), with a low population density compared to similar-sized U.S. metropolitan areas. Vegetated areas are interspersed throughout the suburban areas into the urban core. Properly characterizing the vegetation fraction is critical in simulating Houston’s UHI, as it affects the distribution of surface temperature and possibly extreme heat [47].

### Parcel-level Land Use Data

Parcels are divisions of land areas by individual ownership and can be characterized by land use type. Present-day (2010) and future (2040) parcel-level land use data were acquired from the Houston-Galveston Area Council (H-GAC) [51]. H-GAC is a regional planning organization that provides leadership and guidance in managing change the Houston-Galveston region. H-GAC produces forecasts of populations, employment and land use as part of their quarterly Regional Growth Forecast conducted for the eight counties in the region. Future (2040) parcel-level land use is the product of H-GAC’s Real Estate Development model (http://www.h-gac.com/community/socioeconomic/forecasts/2040/documents/read-documentation.pdf). The model projects future parcel types as a function of planned projects/developments, a parcel’s physical and economic suitability and feasibility for development, and demand for residential housing and non-residential space, which is informed by H-GAC demographic and employment predictive models. Population growth for the Houston-Galveston area is implicit in these models, capturing the population and demographic shifts expected by mid-century in the study area. Land use types for each parcel were assigned by H-GAC and are listed in Table 1. 2040 classifications include two additional categories, “Undetermined” and “Multiple”, where “Undetermined” reflects a parcel that could not be predicted from the model and “Multiple” reflects a parcel that contains more than one of the previous land use types (e.g., combination of residential and commercial). The vegetated proportion for each land use type was estimated via visual comparison of the parcels to imagery from Google Maps. For instance, an H-GAC parcel designated as “residential”, on average, had about 75% vegetated coverage when compared with imagery from Google Maps. These estimates were compared with gridded 1-km vegetation fraction estimates over Houston from the National Urban Database and Access Portal Tool (NUDAPT) [52] and found to be in good agreement.

**Table 1:** Proportion of vegetated land, per H-GAC parcel-level land use type.

Land Use Type	Vegetated Land Proportion
Vacant Developable (includes farming)	1.00
Parks and Open Space	1.00
Undevelopable	1.00
Residential	0.75
Commercial	0.30
Industrial	0.20
Government/Medical/Educational	0.50
Roads	0.00
Water	-
Unknown	-
Other	-
Undetermined (2040 only)	-
Multiple (2040 only)	0.30

Because the shape and size of parcels do not match the shape and size of the cylindrical equidistant 1-km grid (125 x 125 cells) of the climate model domain, the climate data grid was overlaid on the area for which we had H-GAC parcels (henceforth referred to as the “study area”). The parcel level land use data were aggregated to each 1-km grid cell by calculating the proportion of each land use type in each grid cell using ArcGIS 10.2 (ESRI, Redlands, CA). We adjusted the proportion of each land use type so that all proportions in each grid cell added up to 1.0 after removal of the “Other”, “Unknown”, and “Water “land use types for the 2010 and 2040 parcels, as well as the “Undetermined” for 2040; this was done because the LSM only simulates conditions over known land surfaces. Omitting the water proportion may lead to daytime warm biases for grid points near inland water bodies.

The vegetated fraction of a 1-km grid cell was calculated as (Eq 1) the sum of the proportion of each land use type, weighted by proportion of vegetated space listed in Table 1. The urban fraction was the difference of the vegetated fraction from 1.00 (Eq 2).

$$Vegetated\;Fraction=\;\sum_{\text{i}=1}^{n}LU_{i}V_{\text{i}}$$ (1)

Where LU_*i*_ = proportion of 1km grid designated as a specific land use

V_*i*_ = proportion of vegetation per land use

*n* = 15,625 1-km cells

Urban Fraction = 1.00 – Vegetated Fraction (2)

Each 1-km grid cell was then classified into one of the four land use categories required by the modeling framework (described below): “Commercial Urban”, “High Density Urban”, “Low Density Urban”, or “Vegetated”. Classification was based on the dominant land use type of the aggregated 1-km grid cells. “Commercial Urban” grid cells were those in which the combined fraction of the commercial and industrial land use types was greater than the combined fraction of the residential and government/medical/educational land use types. In cases where the combined fraction of residential and government/medical/educational land use types was greater, the cells were categorized as “High Density Urban” if the commercial fraction in the grid cells was >0.1 (i.e., approximating mixed-use housing and commercial neighborhoods), and “Low Density Urban” if the commercial fraction was <0.1 (i.e., approximating suburban neighborhoods). Finally, if the Vegetated Fraction (Eq 1) was greater than the Urban Fraction (Eq 2), the grid cell was re-classified as “Vegetated”. The final present-day land use categories were compared to those in the land use dataset used in Monaghan et al. [47] and found to be in good agreement. The four land use classifications were computed for the present and future HGAC land use scenarios and were used as input variables in the modeling framework described next.

### Modeling Framework

A series of simulation experiments, described below, were conducted over Houston for present day and warming climate scenarios at a spatial resolution of 1-km using the offline version of the Noah Land Surface Model [40] known as the High-Resolution Land Data Assimilation System (HRLDAS; [39]). HRLDAS includes a 1-layer UCM [36] in order to represent the exchanges of heat, momentum and water vapor over urban surfaces. The 2 m air temperature and humidity (two variables required for our analysis) were computed from the HRLDAS output using a diagnostic algorithm described in Monaghan et al. [47]. The urban land use and urban/vegetated fraction of each grid cell was specified from the HGAC urban land use and fraction data that was remapped into the four urban classifications required by HRLDAS, as described above. The remaining model configuration followed Monaghan et al. [47] except that for land use and land fraction inputs we used H-GAC data in place of NUDAPT.

**Fig 1:**
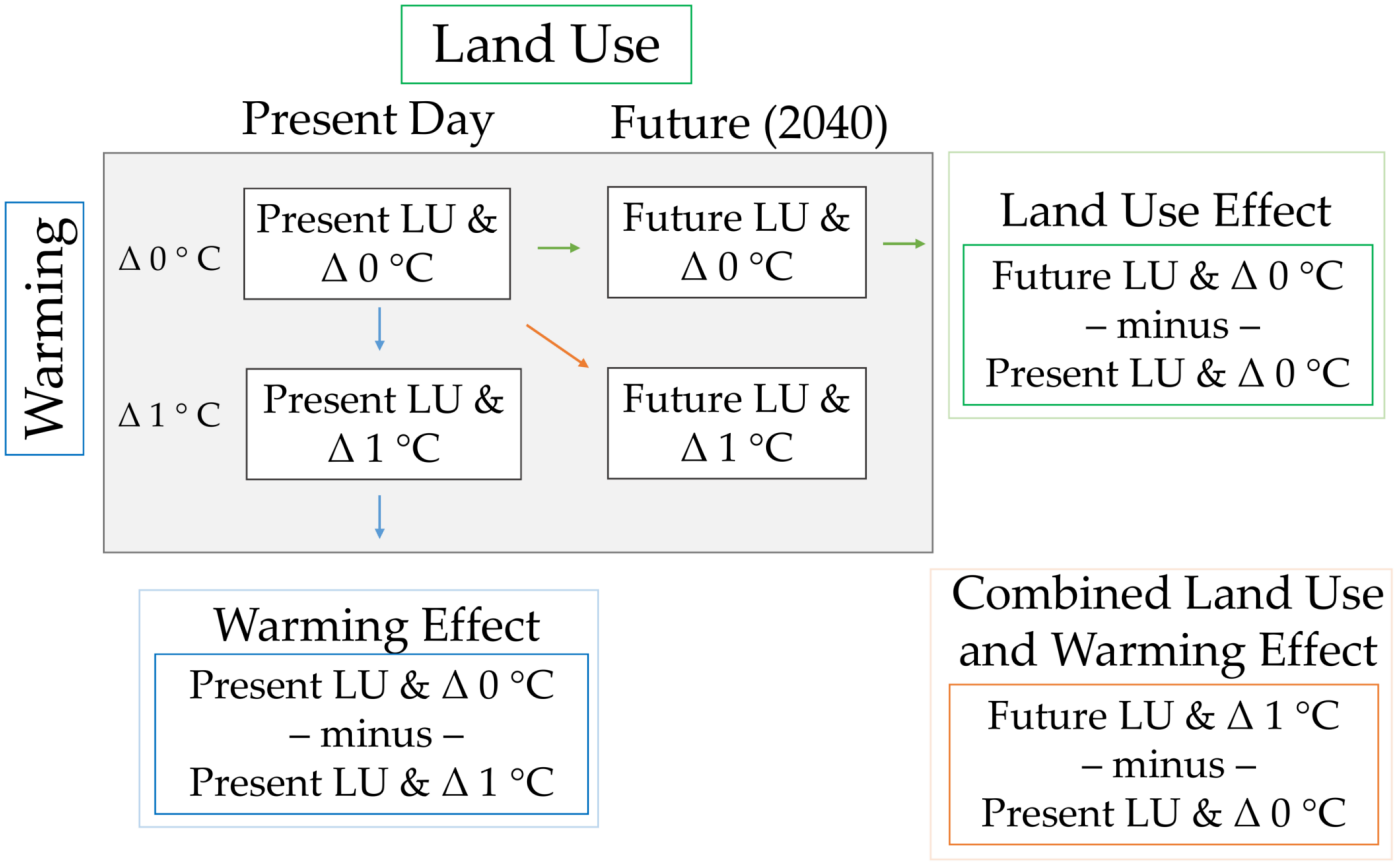
Modeling framework for incorporating present (2010) and future (2040) land use and climate data to estimate the distribution of extreme heat.

Four 10-year-long simulation experiments for were performed with HRLDAS (Fig 1): two for a present day climate scenario and two for a warming scenario. The present-day period, spanning 2003–2012, is approximately centered on the year 2010, and the future period is approximately centered on the year 2040; thus we henceforth refer to these periods as “2010” or “2040”. All four HRLDAS simulation experiments were driven at the upper boundary by hourly meteorological forcing data from phase two of the North American Land Data Assimilation System (NLDAS-2 [53, 54]). The NLDAS-2 forcing data has a spatial resolution of 0.125 degrees and is designed to drive LSMs such as HRLDAS. The two 10-year HRLDAS simulation experiments representing 2010 were driven directly with the 2003–2012 NLDAS-2 forcing data (i.e, no modifications were made to the NLDAS-2 data prior to running HRLDAS). For the two 10-year HLDAS simulation experiments representing 2040, prior to driving HRLDAS three perturbations were made to the 2003–2012 NLDAS-2 forcing data in order to generate the warming scenario. First, the gridded hourly air temperature fields in the 2003–2012 NLDAS-2 forcing data were uniformly increased by 1°C. A value of 1°C is roughly the additional increase in temperature projected for Houston by 2040 [55, 56]. Second, the downward longwave radiation fields in NLDAS-2 were increased by 6 W m^-2^ to be physically consistent with a 1°C temperature increase per the Stefan-Boltzmann Law (i.e., a warmer overlying atmosphere emits more radiation). Third, using the Clausius-Clapeyron equation that relates temperature and humidity, the specific humidity fields NLDAS-2 were increased so as to maintain constant relative humidity between the present-day and warming scenarios. This assumption is consistent with a number of studies indicating that the specific humidity of the atmosphere increases as temperatures increase, but relative humidity generally remains nearly constant [57, 58]. For each pair of present-day and warming simulations, one member used the 2010 HGAC land use data, and the other member used the 2040 HGAC land use data. In this manner, the four simulations provide us with a baseline scenario (2010 HGAC LU and Δ 0°C warming); Experiment 1: a “Warming Effect” only scenario (2010 HGAC LU and Δ 1°C warming); Experiment 2: a “Land Use Effect” scenario (2040 HGAC LU and Δ 0°C warming); and Experiment 3: a “Combined Land Use and Warming Effect” scenario (2040 HGAC LU and Δ 1°C warming). The magnitude of change for each of the three change scenarios is gauged by its difference from the baseline scenario for two variables: daily minimum temperature (T_min_) and daily maximum heat index (HI_max_). The following analysis focuses on the summer months of May-September, when extreme heat events are most likely to occur.

### Threshold Analyses

The NWS issues place-based alerts for excessive heat watches, warnings and advisories based on forecasted heat index values. The NWS’s heat index advisory product communicates to the public the danger to heat exposure at specific heat index values [59]. These are categorized as follows: when the heat index is ≥ 26.7°C (80°F), residents should use *caution*; when the heat index is ≥ 32.8°C (91°F), residents should use *extreme caution*; above 39.4°C (103°F) heat reaches a *danger* level, and heat index values above 52.2°C (126°F) constitute *extreme danger* to humans. Each NWS Forecast Office—there are a 122 nationally—determines the appropriate place-specific threshold for initiating an excessive heat warning or advisory. The Houston-Galveston NWS Office issues a heat advisory when either observed or forecasted temperatures for a county will result in a maximum heat index of 42.2°C (108 °F) or higher for 2 consecutive days.

To evaluate the impacts of land use and warming on the distribution of extreme heat, we considered the number of days that would exceed public health relevant thresholds for both maximum heat index and minimum temperature. We calculated and mapped the average annual number of days that would exceed the heat index thresholds for the NWS’ categorizations of ‘caution’, ‘extreme caution’, ‘danger’, ‘extreme danger’, and the Houston-Galveston NWS Office’s threshold for a heat advisory. The number of days that exceeded the 95^th^ percentile of present-day minimum temperature for the study area was also calculated and mapped.

## Results

### Parcel-Derived Land Use, Present (2010) and Future (2040)

**Fig 2:**
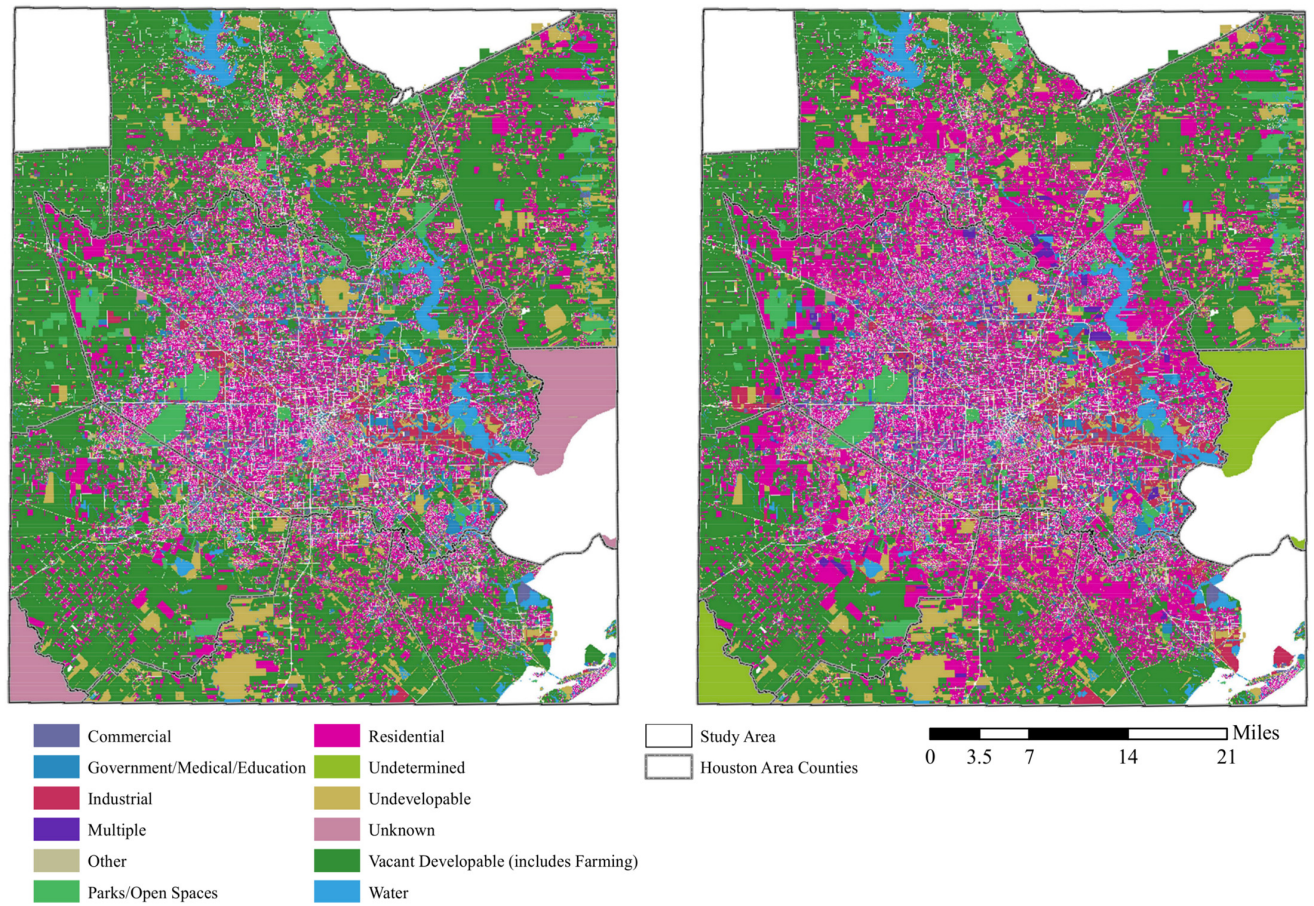
H-GAC land use, by parcel, present (2010; left) and future (2040; right).

**Fig 3:**
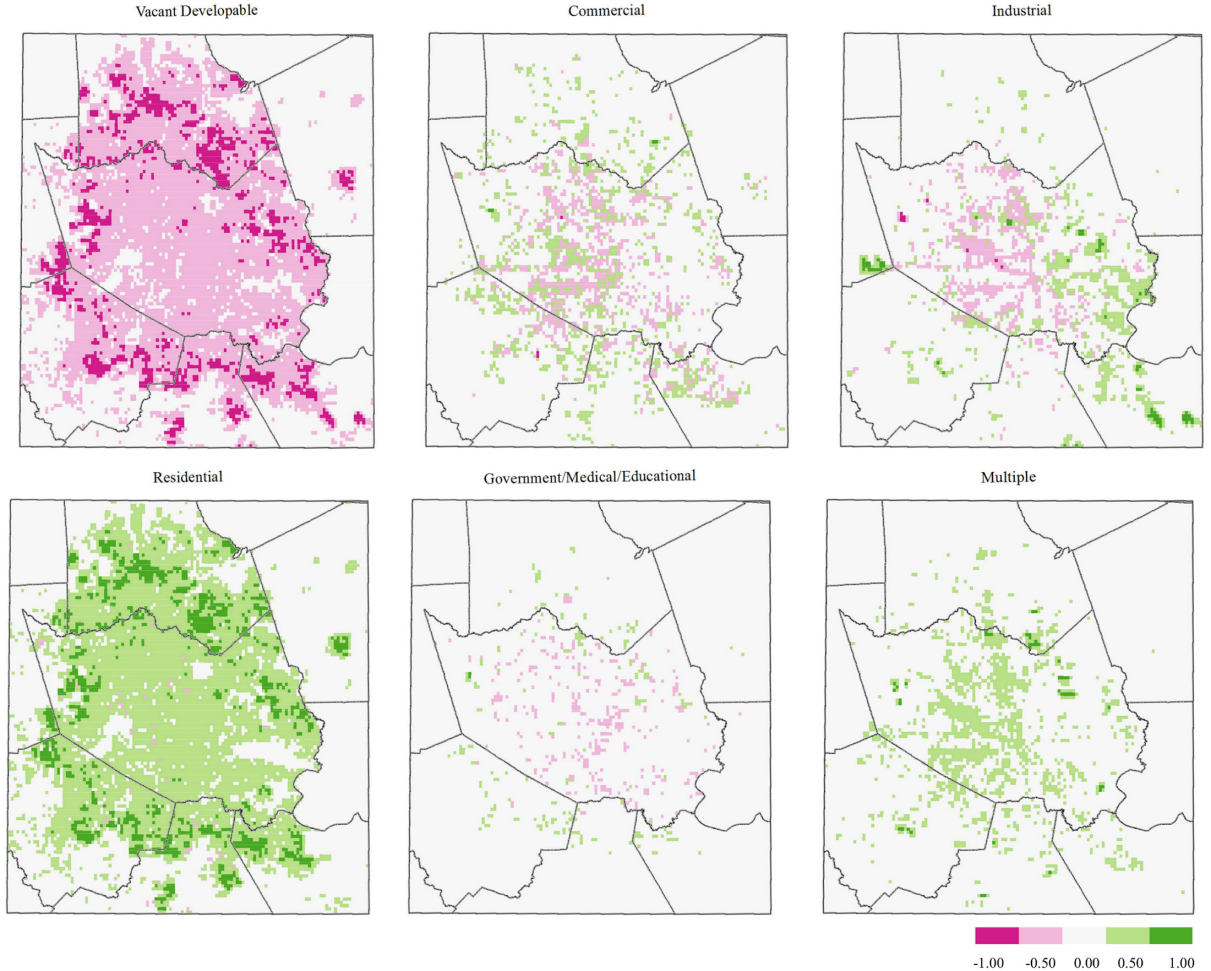
Proportional land use change across the Houston, Texas study area, present (2010) to future (2040).

Visual analysis of the more than two million HGAC parcels in the study area suggested that expected land use change will increase the spatial extent of residential parcels by 2040 (Fig 2). Overall, the surface area of residential land within the study area increased from about 2,600 km^2^ to about 4,100 km^2^; vacant developable land decreased from 6,400 km^2^ to 4,700 km^2^ from the present to 2040. Fig 3 illustrates the change in the HGAC-defined land use between present day and 2040. While the proportion of land cover for most other types remained about the same between the two time periods, there was expected growth in residential and multiple use areas in 2040, with notable decreases in vacant developable land.

The relative change in areal extent of the four remapped urban land use types required by HRLDAS are listed in Table 2. High and low density urban categories saw the largest increase in areal coverage from present day to 2040 of about 30% and 40%, respectively. Predominantly commercial land had the smallest increase in areal coverage, but observed the highest increase in heat index and minimum temperatures in 2040 compared to present day. Higher heat index measurements were observed for future vegetated land, followed by high and low density areas.

**Table 2:** Domain-averaged change in areal extent, HI_max_ and T_min_, between 2010 and 2040 for the four remapped land use type categories used in HRLDAS.

Land Use Type	Ratio of Surface Area Covered by LU Type (2040:Present)	Average Δ HI_max_ (2040 -minus- Present) (°C)	Relative average Δ T_min_ (2040 -minus- Present) (°C)
Commercial	1.18	2.51	1.06
High Density Urban	1.32	2.34	0.99
Low Density Urban	1.41	2.38	0.96
Vegetated	0.82	2.42	0.97

**Fig 4:**
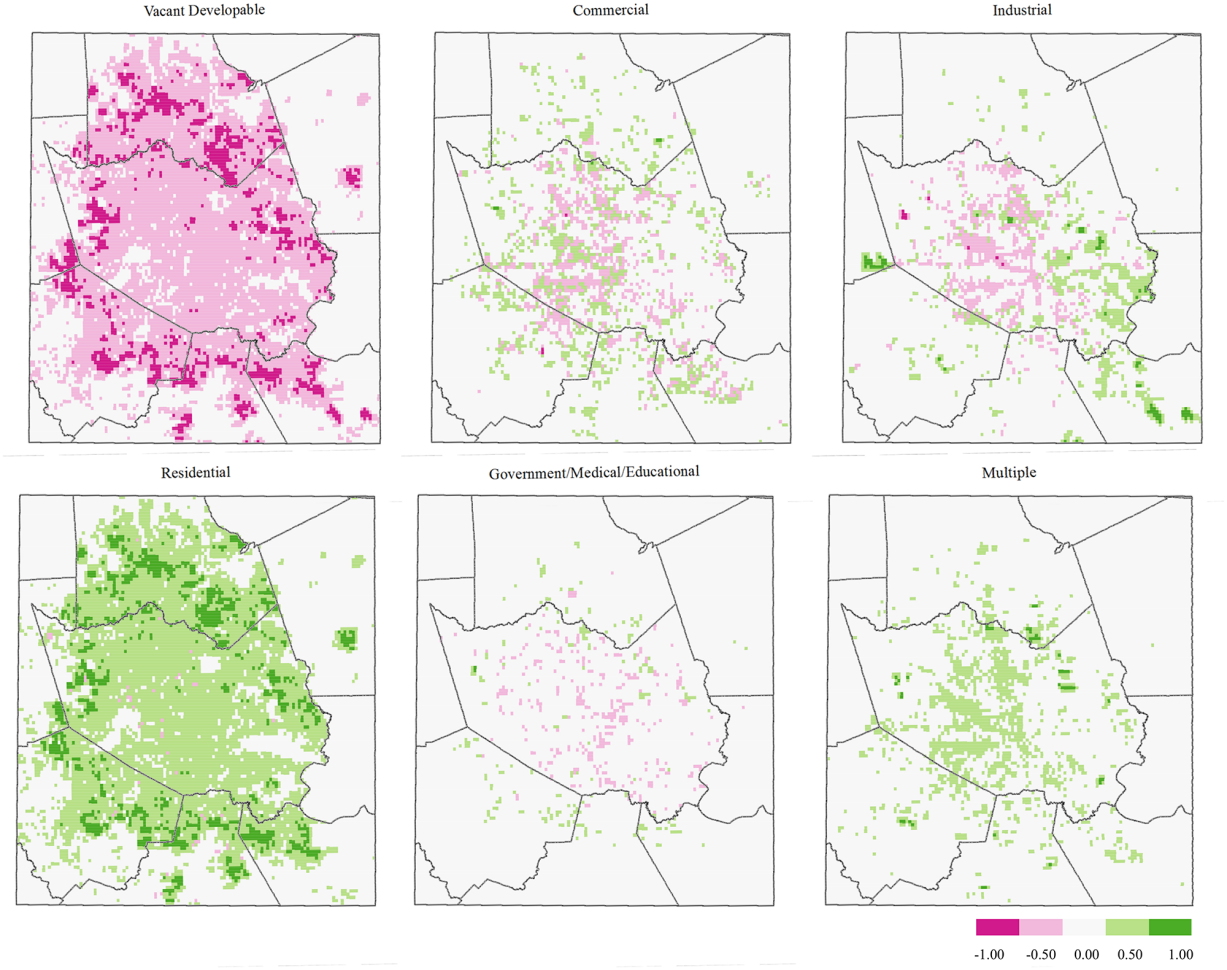
Difference maps (2040–2010), HGAC land use type. Red represents the loss of a specified land use type, whereas green represents a gain.

Fig 4 displays direction of change in the proportion of HGAC land use coverage from 2010 to 2040. Consistent with results shown in Fig 3, vacant developable land—characterized as vegetated space—is projected to decrease by about 20%, with the majority of that land cover type being replaced with residential land use. Patterns of vegetated land loss surrounding the central study area mimic patterns of increased residential land cover. Similarly, the change patterns of commercial land loss match those of industrial and multiple-use land cover increases in the central and eastern portions of the study area.

### Independent and Combined Effects of Land Use and Warming

**Fig 5:**
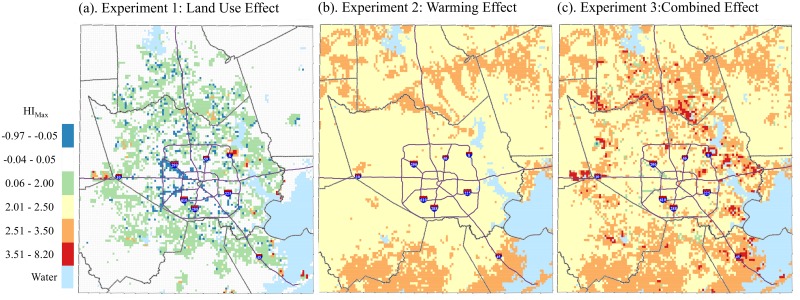
Simulated May-September median T_min_ differences between (a) Future Land Use, Δ 0°C and Present Land Use, Δ 0°C; (b) Present Land Use, Δ 1°C and Present Land Use, Δ 0°C.

**Fig 6:**
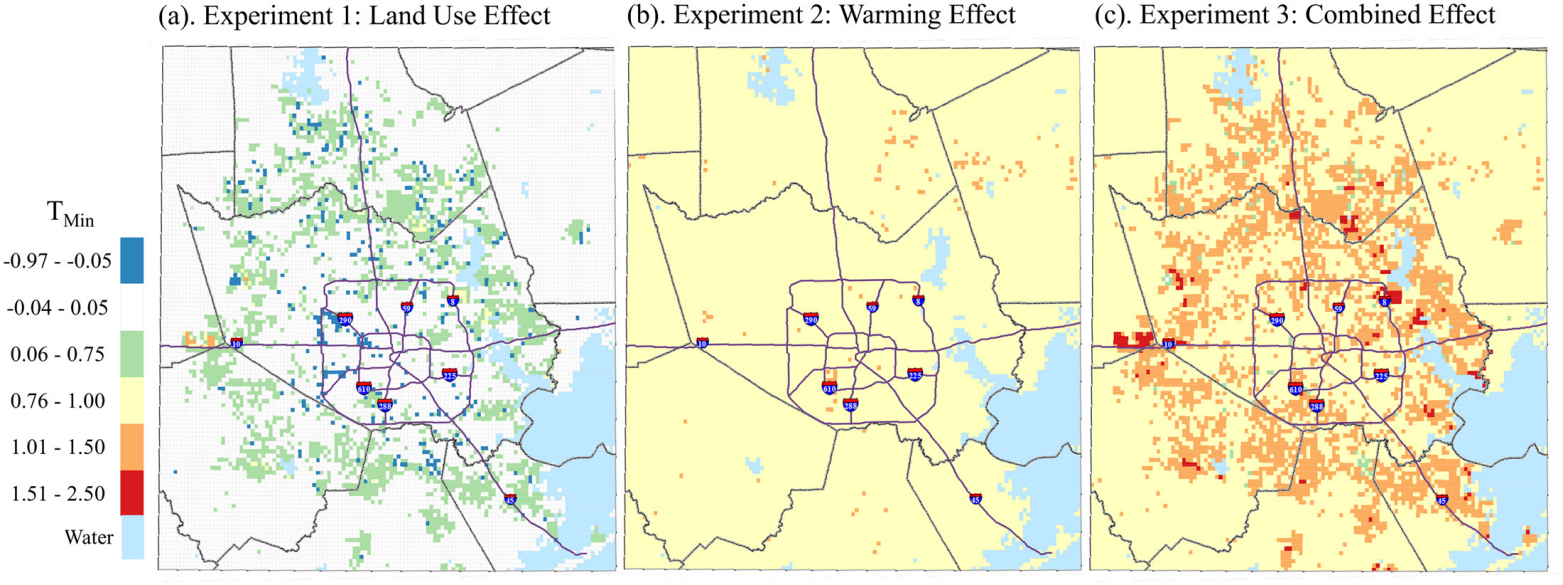
Simulated May-September median HI_max_ differences between (a) Future Land Use, Δ 0°C and Present Land Use, Δ 0°C; (b) Present Land Use, Δ 1°C and Present Land Use, Δ 0°C; (c) Future Land Use, Δ 1°C and Present Land Use, Δ 0°C, 1-km grid, Houston study area.

Both land use and climate change have important impacts on the distribution of T_min_ (Fig 5) and HI_max_ (Fig 6). Simulations for Experiment 2 resulted in patterns comparable to what was seen in the land use change difference maps in Fig 4. Where the residential land use type is projected to increase—the perimeter surrounding the central portion of the study area—we noted increases in T_min_ of about 0.75°C and HI_max_ of about 2°C for the land use effect (Figs 5a and 6a). There is a slight cooling effect in some areas within the city where commercial and industrial land is projected to be converted to other less developed urban land use categories such as multiple.

In Experiment 2, where a 1°C increase was added to the forcing data in HRLDAS while maintaining present day land use, we noted a steady warming of 1.0°C for T_min_ and ~2–2.5°C for HI_max_ across the study area (Figs 5b and 6b). Experiment 3 simulations yield strong warming nearly everywhere (T_min_ from ~1.5–2.5°C and HI_max_ from ~3.5–8.3°C), particularly where land use is projected to be converted from vacant into residential, industrial and multiple land use types (Figs 5c and 6c). Spatial patterns for both T_min_ and HI_max_ maps were similar, with the largest increases occurring along the newly developed perimeter of the city. Notable pockets of increased temperature also occur along the coast and the eastern ship channel.

### Implications for Public Health

Calculations for days exceeding public health-relevant thresholds for the combined land use and warming scenario are presented in Table 3. Days in 2040 exceeding the 2010 summertime 95^th^ percentile of T_min_ increased by 357%-416% across the study area and are projected to occur on about 22–25% of nights depending on the land use type; the increases were progressively larger for more developed urban land use types. The NWS general heat advisory products based on HI_max_ shifted from the lower categories in 2010 (‘caution’ and ‘extreme caution’) to more severe categories in 2040 (‘danger’ and ‘extreme danger’). Days exceeding the Houston-Galveston area NWS threshold of two consecutive days of HI_max_ above 42°C also increased substantially by 2040. On average across the study area, a nearly four-fold increase in the number of days exceeding the threshold for heat index values above 42°C for two days is projected for 2040; about 19% of days are projected to exceed the threshold for 2040, whereas just 4% of days exceed the threshold for present day. The commercial urban areas are projected to have the largest increase in number of days exceeding this threshold (31.4 per year), mainly because these areas are already near the threshold to begin with due to their heat-retaining thermal properties. Though vegetated areas are projected to have the smallest increase in the number of days exceeding this threshold (18.0 per year), they exhibit the largest percentage increase (462%) because comparatively few days exceed the threshold during present day. Vegetated/rural areas in this region often stay cooler than urban areas because they have more near-surface moisture (i.e., in soils) than urban areas and thus more energy can be partitioned toward evaporation rather than raising temperatures.

**Table 3:** Comparison of results for seven public-relevant thresholds for two scenarios. The two scenarios are 1) present land use and no warming, and 2) 2040 land use with +1°C warming. The results are presented according to the land use type, whereby the values are calculated by averaging across all grid points of a specified land use type for a given scenario. Columns 1–2 and 3–4: for each scenario, the annual average number of summer days exceeding the threshold and the threshold at which exceedance occurs; Column 5: the number of excess days in 2040 compared to present day; Column 6: the percent change of days above the threshold in 2040 compared to present day. The results are organized by HRLDAS-specific land use types. T_min_ = Minimum daily temperature; HI_max_ = Maximum daily heat index.

	Scenario				Above Threshold	
Exposure Threshold	Present Land Use, Δ 0°C	Percentile	2040 Land Use, Δ 1°C	Percentile	Excess Days (*n*)	% Δ (2040—Present)
T_Min_						
Total Study Area	7.5	95	35.5	77	28.0	373%
Commercial Urban	7.5	95	38.7	75	31.2	416%
High Density Urban	7.5	95	37.6	75	30.1	401%
Low Density Urban	7.5	95	35.8	77	28.3	373%
Vegetated	7.5	95	34.3	78	26.8	357%
HI_Max_, 2 Days Above 42°C						
Total Study Area	6.3	96	29.4	81	23.1	367%
Commercial Urban	17.4	89	48.8	68	31.4	181%
High Density Urban	6.8	96	32.4	79	25.6	377%
Low Density Urban	7.8	95	34.6	77	26.8	344%
Vegetated	3.9	98	21.9	86	18.0	462%
HI_Max_, NWS "Caution"						
Total Study Area	16.8	89	9.9	94	-6.9	-41%
Commercial Urban	11.5	93	6.9	96	-4.6	-40%
High Density Urban	14.6	90	9.1	94	-5.5	-38%
Low Density Urban	14.4	91	8.8	94	-5.6	-39%
Vegetated	18.1	88	11.2	93	-6.9	-38%
HI_Max_, NWS "Extreme Caution"						
Total Study Area	87.9	43	59.6	61	-28.3	-32%
Commercial Urban	73.0	52	45.4	70	-27.6	-38%
High Density Urban	84.3	45	57.2	63	-27.1	-32%
Low Density Urban	83.0	46	55.7	64	-27.5	-33%
Vegetated	91.7	40	65.0	58	26.7	29%
HI_Max_, NWS "Danger"						
Total Study Area	46.1	70	81.1	47	35	76%
Commercial Urban	67.3	56	99.8	35	32.5	48%
High Density Urban	52.2	66	84.7	45	32.5	62%
Low Density Urban	53.7	65	86.8	43	33.1	62%
Vegetated	40.5	74	74.1	52	33.6	83%
HI_Max_, NWS "Extreme Danger"						
Total Study Area	0.8	99.5	1.3	99.2	0.5	63%
Commercial Urban	0.2	99.9	0.3	99.8	0.1	50%
High Density Urban	0.5	99.7	1.1	99.3	0.6	120%
Low Density Urban	0.5	99.7	0.9	99.4	0.4	80%
Vegetated	0.9	99.4	1.5	99.0	0.6	67%

**Fig 7:**
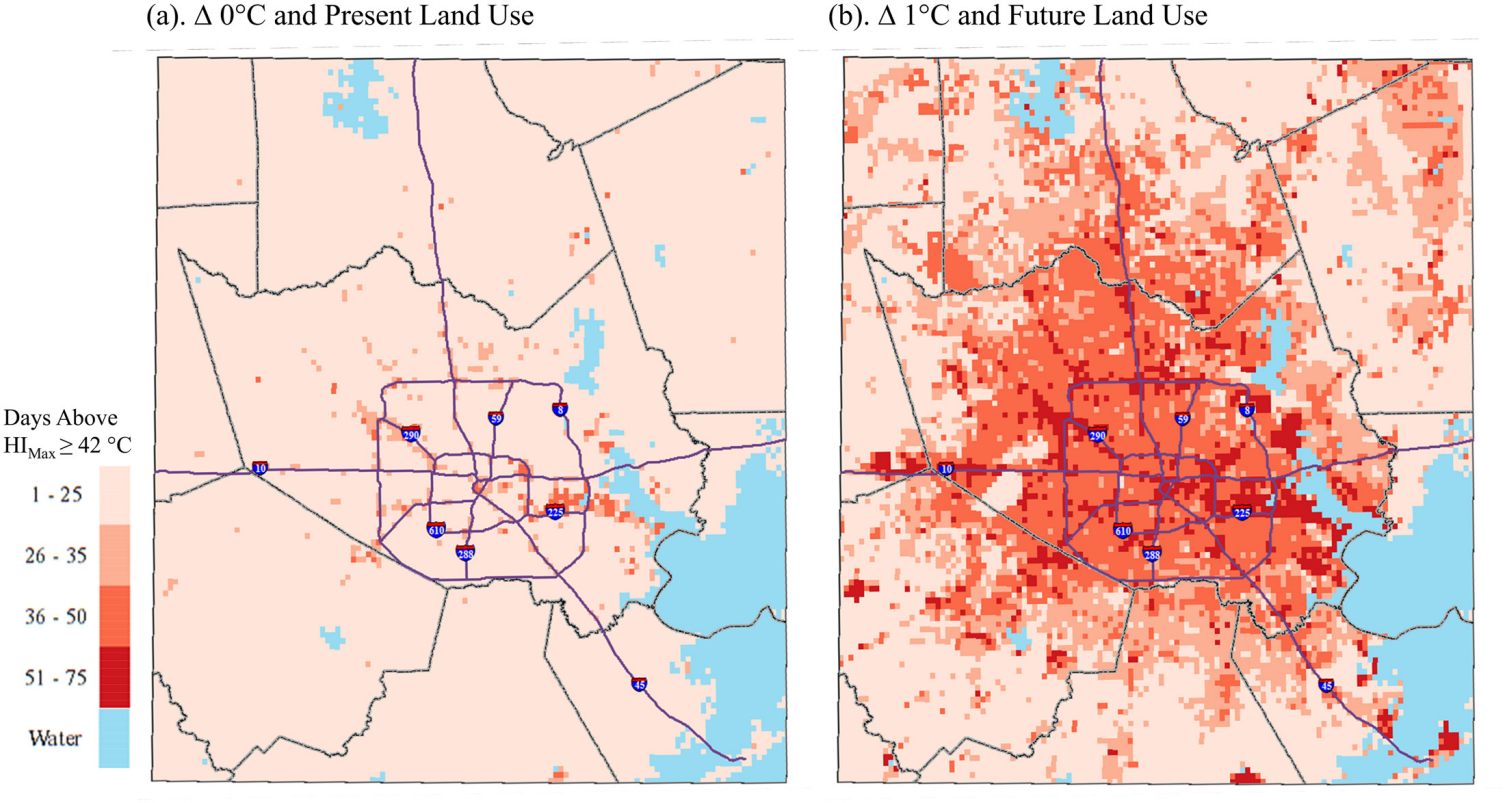
Average annual number of May-September days exceeding the NWS threshold (two consecutive days with HI_max_ > 42°C) for initiating a heat advisory for (a) the baseline scenario and (b) the combined climate and land use change scenario, 1-km grid, Houston study area.

**Fig 8:**
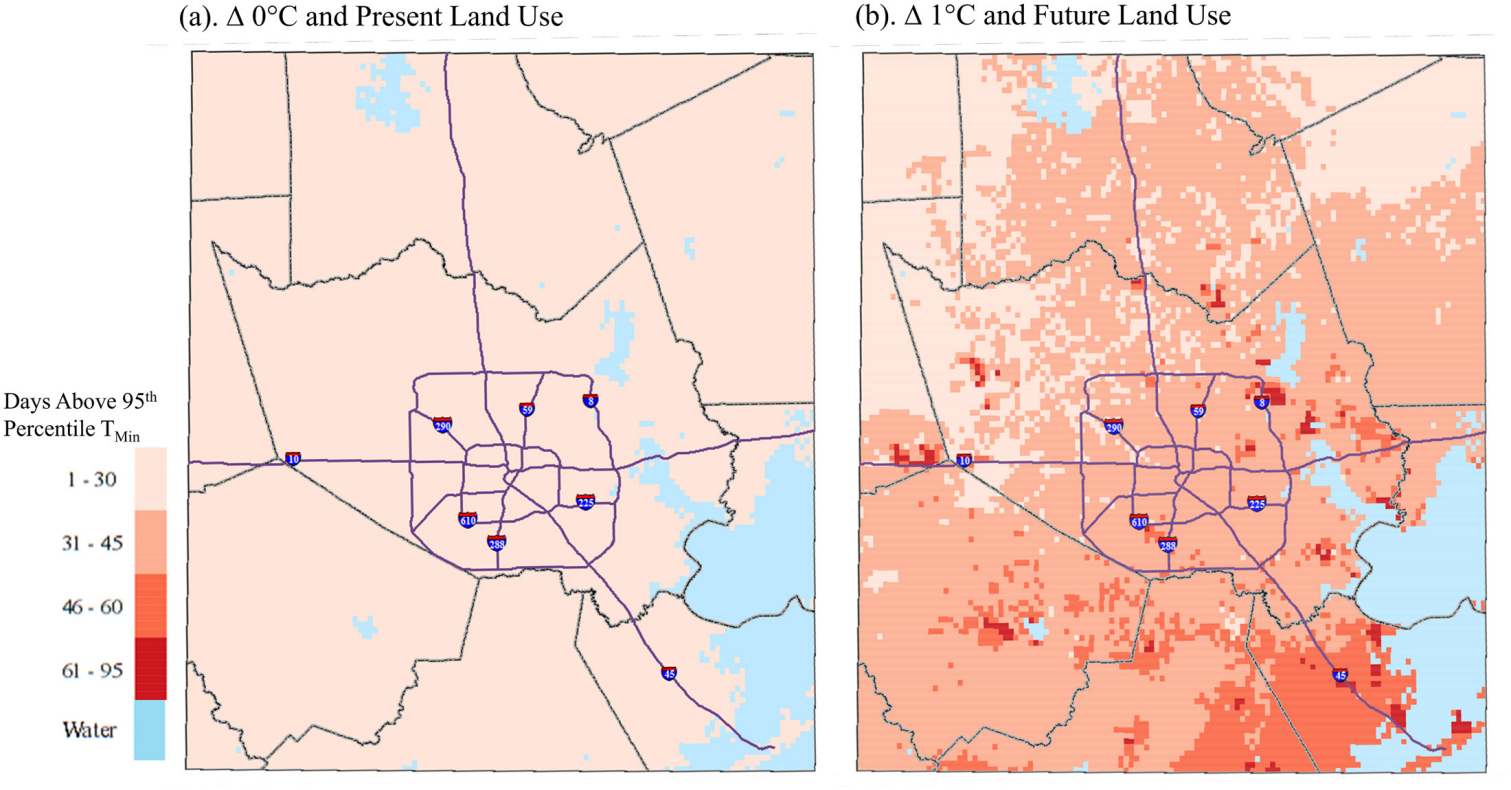
Average annual number of May-September days exceeding the 95^th^ Percentile of T_min_ for (a) the baseline scenario and (b) the combined climate and land use change scenario, 1-km grid, Houston study area.

In addition to the study area-wide totals shown in Table 3, the intra-urban distribution of two important thresholds is shown in Figs 7 and 8 for the baseline scenario and the combined climate and land use change scenario. The spatial pattern of the number of May-September days of HI_max_ > 42°C for the baseline (2010) scenario indicates the greatest exceedances along major thoroughfares and the Houston ship channel adjacent to the eastern coast (Fig 7a). The number of days above the baseline (2010) 95^th^ percentile of T_min_ is, by definition, constant across the study area equal to 7.5 days; i.e., 5% of the 153 total days from May-September (Fig 8a). When imposing combined climate and land use changes for 2040, the spatial distribution of extreme heat changed substantially. A widespread increase in average number of summer days above the NWS threshold for heat index values was evident in central, northern and coastal portions of the study area (Fig 7b). On average, and mostly along thoroughfares and in the coastal areas, about 42% of days during summer months would be expected to exceed the NWS threshold for a heat advisory. Minimum temperature changes by 2040 were more uniform, though pockets of significantly higher minimum temperatures would be observed in coastal and western pockets of the study area—notably where there is projected conversion to the industrial land use type—for >50% of summer days.

## Discussion

We evaluated the individual and combined impacts of land use and climate change on the intra-urban distribution of heat for Houston, Texas between 2010 and 2040. Using planned land use changes for the Houston area for 2040, present-day and future simulations of T_min_ and HI_max_ indicated that modifications to the urban environment may substantially influence the distribution of heat across Houston. By 2040, the loss of predominantly vegetated vacant developable land appeared to be converted mainly to residential land; patterns of commercial and industrial land cover loss match gains in multiple-use land cover. The increased development alone added to increases in T_min_ and HI_max_; in some cases where predominantly vacant (vegetated) land was converted primarily to commercial and industrial uses, the temperature increases due to land use change exceeded those due to warming. Zhou et al [60] reported similar effects of increased minimum temperatures in Houston’s high density urban areas. Simulations in which we held land use constant at 2010 definitions but allowed temperatures to increase by 1°C according to 2040 climate projections for Houston indicated relatively uniform increases in T_min_ and HI_max_ across the study area, as expected, given the slowly varying (in space) nature of temperature changes [61]. Thus, the combined effects of land use change and climate change may alter the spatio-temporal variability of populations exposed to extreme heat. This finding corroborates another Houston-specific study that identified urban and rural areas as being at-risk for increased future exposure to high daytime and nighttime temperatures [49]. Our experiments indicate higher T_min_ and HI_max_ along the present-day urban perimeter where increases in residential, industrial, and multiple land use types are projected for 2040. An approximate four-fold increase in the average annual number of days that exceed the present day NWS threshold for a heat advisory for the general Houston study area could potentially expose many more people to extreme heat.

A unique outcome of these analyses was the change in the distribution of days exceeding NWS heat advisory thresholds for the Houston area by 2040 compared to 2010 for the combined land use and climate change scenario. The number of days in the NWS “caution” and “extreme caution” categories declined. However, the number of days that fell into the higher tier categories of “danger” and “extreme danger” increased. Thus, the days lost to the lower threshold categories were reallocated to the higher threshold categories. Further, in all advisory categories, locations characterized as primarily vegetated land use types in 2010 would have two- to three-times the number of days exceeding the heat advisory threshold in 2040. These results suggest that a large portion of the population living in the Houston study area, presumably in residential, mixed-used, low-, and high-density urban neighborhoods, may face large increases in the number of summer days where temperatures will meet the criteria for a NWS heat advisory. Similarly, even areas categorized as primarily vegetated may see more days above NWS advisory thresholds—a result consistent with the general shift.

The efficacy of the Houston area advisories in preventing heat-related morbidity and mortality has not been formally evaluated, though, understanding impacts on public health via population exposure modeling could inform advisory and warning criteria. In Houston, higher nighttime temperatures are associated with increased mortality [25]. Lower nighttime temperatures provide respite from high daytime temperatures, which is particularly important during periods of prolonged high temperatures [23, 62]. Our results suggest that higher minimum temperatures—a signature of the UHI—will become a growing concern for population health in Houston. Heat warning and advisory criteria could be further evaluated in anticipation of the combined effects of climate and land use changes.

Efforts to reduce population exposure to extreme heat fall along a spectrum from individual-level behavior modification to community-level built environment alterations. Urban design can introduce complexities that influence microclimates, such as surface structure and housing density moderating fine-scale ambient temperatures [63, 64]. Modeling studies have shown that UHI mitigation efforts extending beyond the urban core, such as increased tree canopy in suburban areas, can reduce the UHI effect [65]. High density urban cores, however, may expose larger residential populations to extreme heat during heat waves as shown by Adachi et al [41]. Thus, tradeoffs exist between the benefits of higher residential urban density and increased suburban vegetation. In a city like Houston, where there are no zoning laws [66], planners and developers can maximize vegetation within development projects. According to a survey of Houstonians by the Kinder Institute [67], current residents are pleased with the direction of the city’s development, though more than half would prefer “smaller homes in more urbanized areas”. Multiple-use parcels are expected to increase by mid-century in Houston and could serve as an example for other similarly developing cities.

The study presented here is limited in its ability to project precise population exposure to extreme heat as a function of land use and climate change. The population for the Houston metropolitan region was slightly less than six million in 2010, and under moderate demographic growth scenarios is expected to add roughly 3.2 million by 2040 [68]. Incorporating demographic shifts and substantial population growth can strengthen estimates to determine which populations will be vulnerable to extreme heat. Although population growth is implicit in the parcel-level land use model, it is nearly impossible to predict how demographics will change at such a fine scale. Despite this limitation, the land use model and its outputs are useful in guiding physical modifications that can be executed via climate change adaptation policies. An additional limitation is that the use of the offline HRLDAS climate model simulations did not allow for non-linear interactions between the land surface and atmosphere, which may impact the overall change in T_min_ and HI_max_ in unknown ways. Further, the experiments presented here do not account for the full range of potential climate changes, such as changes in precipitation and cloud cover that may modulate extreme heat.

Accurately projecting heat-related health impacts requires place-specific knowledge of historical temperature-mortality relationships and future projections of climate and environmental variables, population changes and population acclimatization [6]. In part, the exposure-response model of heat and adverse health is sensitive to modeled exposure variables [69]. The objective of this study was to demonstrate the impact of climate change and fine-scale land use modifications on Houston’s present and future UHI through use of a simple, off-line land surface model. Our findings indicate that high-resolution models that incorporate climate change and land use change may be useful for projecting future intra-urban exposure to extreme heat. Such models could be utilized to inform urban planning and public health programmatic and policy development aimed to reduce population exposure to heat.
