# Correction: Potential Impacts of Future Warming and Land Use Changes on Intra-Urban Heat Exposure in Houston, Texas

**DOI:** 10.1371/journal.pone.0151226

**Published:** 2016-03-18

**Authors:** Kathryn Conlon, Andrew Monaghan, Mary Hayden, Olga Wilhelmi

Figs 3 and 4 are duplicates; the correct Fig 3 is included below. Figs 5 and 6 are out of order and do not correspond to the correct figure captions. Please view Figs 5 and 6 in the correct order below.

**Fig 3:**
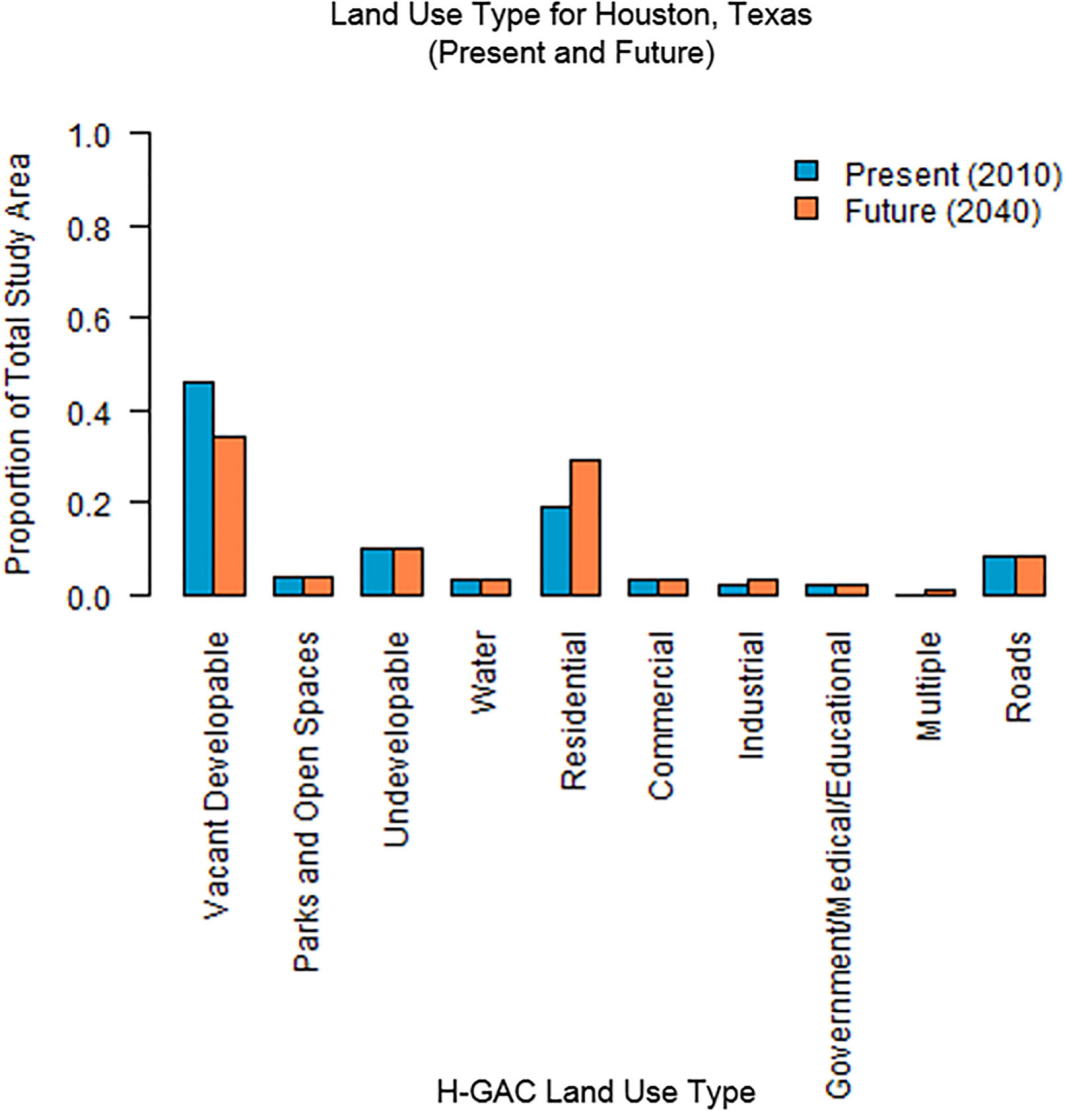
Proportional land use change across Houston, Texas study area, present (2010) to future (2040).

**Fig 4:**
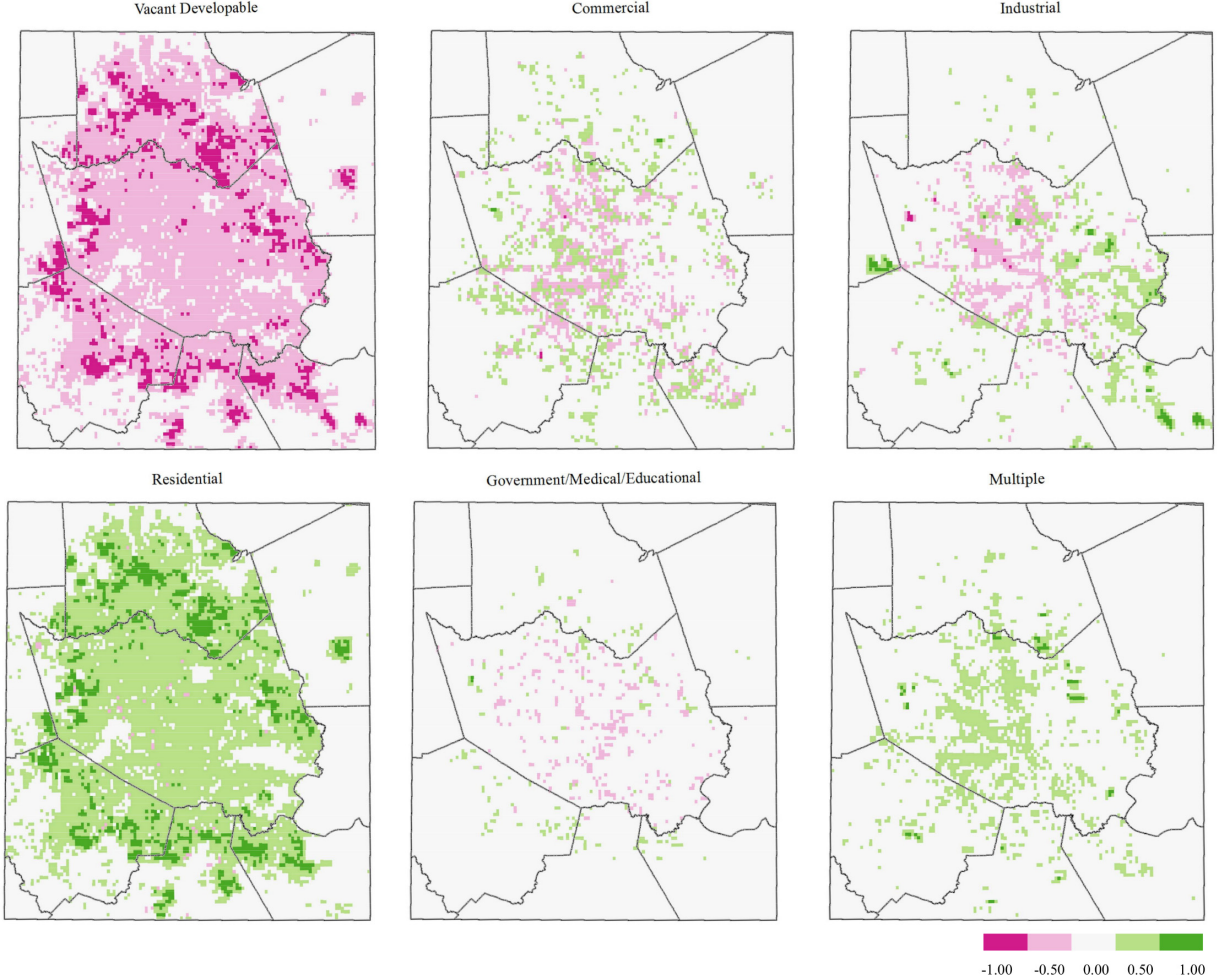
Difference maps (2040–2010), HGAC land use type. Red represents the loss of a specified land use type, whereas green represents a gain.

**Fig 5:**
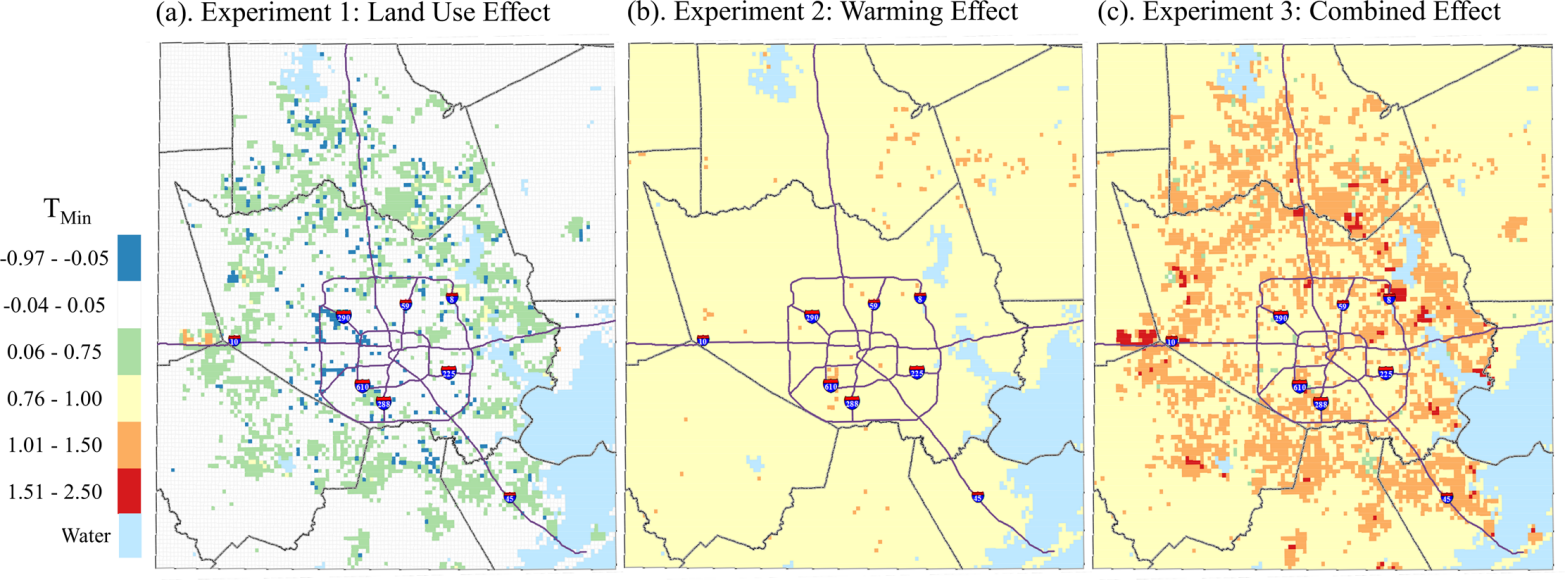
Simulated May-September median T_min_ differences between (a) Future Land Use, Δ 0°C and Present Land Use, Δ 0°C; (b) Present Land Use, Δ 1°C and Present Land Use, Δ 0°C; (c) Future Land Use, Δ 1°C and Present Land Use, Δ 0°C, 1-km grid, Houston study area.

**Fig 6:**
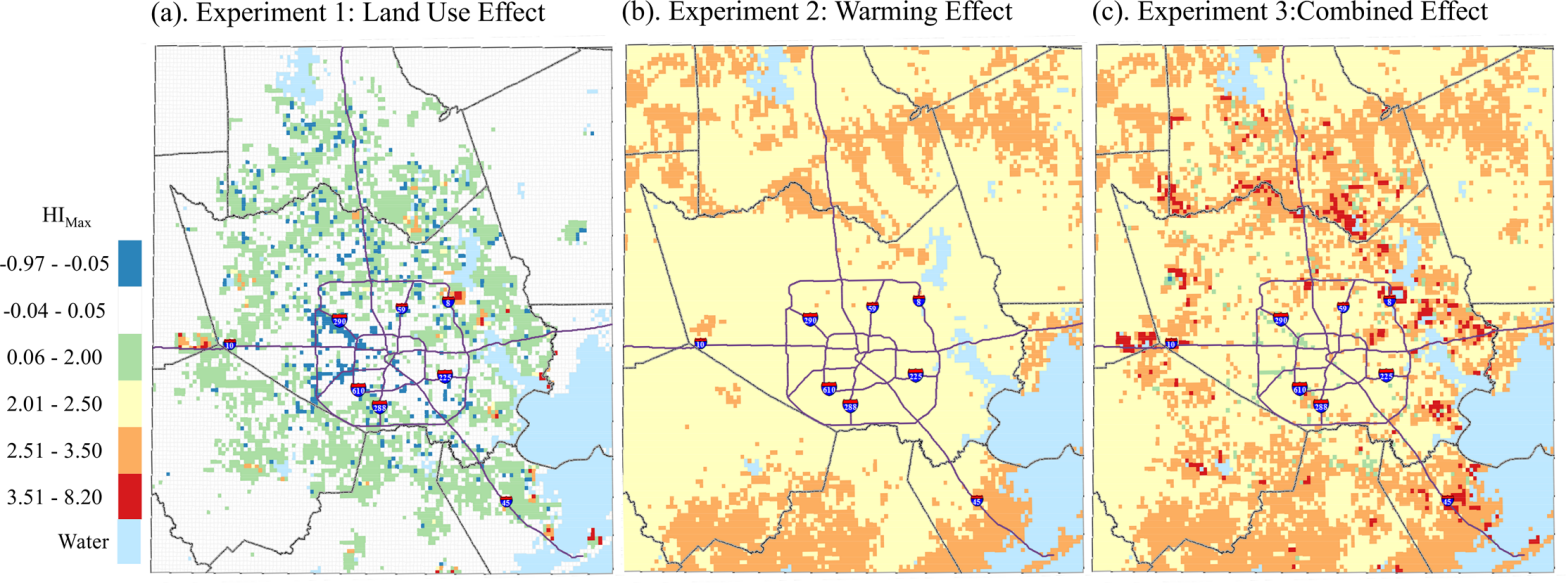
Simulated May-September median HI_max_ differences between (a) Future Land Use, Δ 0°C and Present Land Use, Δ 0°C; (b) Present Land Use, Δ 1°C and Present Land Use, Δ 0°C; (c) Future Land Use, Δ 1°C and Present Land Use, Δ 0°C, 1-km grid, Houston study area.
